# Anteromedial cannulated screw fixation for Hawkins II/III talus fractures in children: a retrospective study

**DOI:** 10.1186/s13018-023-04253-y

**Published:** 2023-10-10

**Authors:** Xincheng Huang, Siyuan Ruan, Zhuolin Lei, Hong Cao

**Affiliations:** Department of Traumatic Orthopedics, Renmin Hospital, Hubei University of Medicine, Shiyan, 442000 People’s Republic of China

**Keywords:** Anteromedial cannulated, Hawkins II/III, Talus fractures, Children

## Abstract

**Objective:**

To investigate the clinical effect of the anteromedial cannulated screw approach in the treatment of Hawkins II/III talus fractures in children.

**Methods:**

A retrospective study was conducted on 20 children with talar fractures admitted to Renmin Hospital from September 2018 to February 2022. The fracture healing and functional recovery of the affected limb were strictly followed up after the operation. There were 14 males and 6 females. The average age was 9 years (range 6–12 years). According to the Hawkins classification, there were 12 cases of talar neck fracture type II and 8 cases of type III. All patients were fixed with cannulated compression screws via an anteromedial approach. According to the American Orthopedic Foot and Ankle Society ankle and hindfoot function scoring system, limb function was evaluated before and after the operation. A visual analog scale was used to evaluate the degree of postoperative pain.

**Results:**

All 20 children were followed up for 12 months to 30 months, with an average of 15 months. We found that there was no significant difference in the excellent and good rate (76.9%) and necrosis rate (30.8%) between male children and female children (71.4%) and necrosis rate (28.6%) (*P* > 0.05). The excellent and good rates (92.9%) of children younger than 9 years old at the time of injury were higher than those of children older than 9 years old (33.3%), and the incidence of avascular necrosis of the talus was lower. The differences between the two groups were statistically significant (*P* < 0.05). The average prognosis score of children who underwent surgery within 5 days after injury was 89.2 ± 6.4, which was significantly higher than that of children who underwent surgery after 5 days (72.9 ± 13.1), and the difference was statistically significant (*P* < 0.05). There was no significant difference between patients who underwent surgery within 5 days after injury (15.4%) and those who underwent surgery after 5 days (51.7%) (*P* > 0.05). The excellent and good rates of talar neck fracture type II and talar neck fracture type III were 90.1% and 55.6%, respectively.

**Conclusion:**

The anteromedial approach combined with cannulated compression screws for the treatment of Hawkins II/III talus fractures in children not only has a clear surgical field, but the fracture can also be reduced and fixed under direct vision using this technique. It does not affect the stability of the ankle joint and is conducive to the recovery of ankle function. It can be used as a surgical scheme for the treatment of talar fractures in children.

## Introduction

Talus fractures are rare in children, with a reported incidence of 0.01% to 0.08% [1, 2]. The main clinical manifestations are obvious swelling and pain in the ankle after fracture, which seriously affect the functional activities of the ankle joint. Although the incidence of talus fracture in all children is extremely low [3], postfracture management is difficult, and various complications often occur due to improper treatment due to the features of irregular talus morphology, more articular cartilage coverage, less blood supply and a complex relationship with adjacent joints [4], for example, ischemic necrosis of the talar body, skin infection necrosis, traumatic osteoarthritis, malunion and so on [5]. The main damage is the blood supply outside the bone and the blood vessels inside the bone, especially when the fracture is displaced, and the necrosis rate is as high as 70–100% [6]. This can seriously affect ankle function, especially in children with a longer life expectancy [7]. According to the anatomical characteristics of the talus and the location of fracture, talar fractures can be divided into talar bone, talar neck and talar body fractures. As with adults, the most common type of talus fracture in children is talus neck fracture, with an incidence of approximately 50%. The Hawkins classification is the most commonly used clinical classification of talar neck fractures, which can be mainly be divided into types I, II, III and IV according to the degree of fracture displacement and dislocation [8]. The treatment and prognosis of fractures in different parts, degrees and ages vary greatly. Due to the low incidence of this disease, there is still a lack of relatively recognized treatment standards, and the influence of various clinical factors on fracture prognosis has not been fully determined. Therefore, to explore the clinical efficacy of anteromedial cannulated screw approach in the treatment of talus fractures in children, further increase the understanding of talar fractures, improve the treatment of this disease, and find better treatment methods, 20 cases of talar fractures in children admitted to our hospital from February 2018 to February 2022 were retrospectively analyzed.

## Materials and methods

### Inclusion and exclusion criteria

The study protocol was approved by the ethics committee of Renmin Hospital Affiliated with Hubei Medical University. Inclusion criteria: ① The age range was 6–12 years, with an average age of 9 years; ② According to the Hawkins talus fracture classification, all were talus fractures of type II/III; ③ Fracture displacement of more than 2 mm that required open reduction and internal fixation; ④ The patient had no congenital heart disease, pulmonary disease, or severe mental or nervous system disease, and could tolerate surgery; ⑤ Complete case data. Exclusion criteria: ① Pathology, open talus fractures or severely contaminated wounds; ② Complications with other severe fractures (examples include calcaneal fractures and distal tibial fractures); ③ Severe heart, lung, and brain diseases, including congenital diseases; ④ Clear surgical contraindications; ⑤ Incomplete medical records or a short postoperative follow-up time. From September 2018 to February 2022, we conducted a retrospective study of 20 pediatric talus fracture patients in Hawkins II/III who met the inclusion criteria in our hospital. There were 14 males and 6 females. The average age was 9 years (range 6–12 years). On admission, all patients underwent foot anteroposterior and lateral X-ray films and CT examination to confirm the classification and to fully understand the anatomical changes in the talus. According to the Hawkins classification, there were 12 cases of type II and 8 cases of type III injuries. The causes of injury included falling from height in 12 cases, traffic accidents in 6 cases and other causes in 2 cases. All patients were followed for at least 1 year (Table 1).

**Table 1:** Basic patient information

	Age (year)	Gender	Injury type	Hawkins types	Complication
1	8	Male	Bruise	III	Yes
2	6	Male	Tumbling down	II	None
3	7	Female	Traffic accident	II	None
4	7	Male	Tumbling down	II	None
5	5	Female	Tumbling down	III	Yes
6	7	Male	Traffic accident	II	None
7	8	Male	Tumbling down	III	None
8	7	Male	Tumbling down	II	None
9	6	Male	Tumbling down	III	None
10	9	Female	Tumbling down	II	None
11	7	Male	Traffic accident	II	None
12	7	Male	Bruise	III	None
13	8	Female	Tumbling down	III	Yes
14	6	Male	Traffic accident	II	None
15	10	Male	Tumbling down	III	Yes
16	11	Female	Traffic accident	III	None
17	10	Female	Tumbling down	III	None
18	12	Male	Tumbling down	II	Yes
19	11	Male	Traffic accident	II	Yes
20	12	Female	Tumbling down	II	None

### Treatment methods

The patients were admitted to the hospital after diagnosis. The relevant examinations were completed first, such as routine blood tests, liver and kidney function tests, and coagulation function tests, and then preoperative X-ray film and CT scan (including trauma color film) of the affected foot were routinely performed. X-ray film and CT showed that the talar cortical bone continuity was destroyed and that the tibiotalar joint was dislocated (including subluxation). Thirteen cases were treated with temporary plaster external fixation of the ankle joint. The preoperative limb function was evaluated according to the American Orthopedic Foot and Ankle Society (AOFAS) ankle and hindfoot function scoring system. All patients were given symptomatic treatment, such as detumescence and pain relief, and surgical treatment was performed after detumescence. A visual analog scale (VAS) was used to evaluate the degree of postoperative pain. All patients were treated with open reduction and internal fixation with cannulated compression screws via an anteromedial approach.

### Operation strategy

After successful anesthesia, the patient was placed in the supine position, an inflatable tourniquet was applied to the upper end of the affected limb, and routine disinfection and drape placement were performed. All patients were treated by an anteromedial surgical approach, and the skin, subcutaneous tissue and deep fascia were dissected. A medial malleolus osteotomy could be performed according to the surgical requirements. After exposing the fracture end, the soft tissue around the talus was retained as much as possible, the joint cavity was cleaned, the detached bone and cartilage fragments were removed, the large cartilage injury was drilled and scratched in the injury area, the fracture fragment was reduced, and the fracture fragment was fixed temporarily with 2 fine Kirschner wires. Two guide pins were inserted along the longitudinal axis of the talus, and 1 controllable compression hollow screw (the number of screws may be increased as appropriate) was inserted after drilling depth. The end of the nail was buried under the chondral surface of the talus. After reduction in the talus, the subtalar joint was well reduced, and the periankle fracture and the osteotomy fragment of the medial malleolus were fixed. C-arm fluoroscopy showed that the fracture was reduced, and the position of the internal fixator was satisfactory. Finally, the wound was closed, and the dressing was pressurized.

### Postoperative treatment

All children were treated with ankle plaster fixation in the neutral position for 6–8 weeks. Regular follow-up was conducted at 1 month, 3 months, 6 months, 1 year and 2 years after the operation for foot pain, joint mobility, walking and gait, fracture healing and talar necrosis. According to the American Orthopedic Foot and Ankle Society (AOFAS) ankle and hindfoot function scoring system, postoperative limb function was evaluated (excellent: 90–100 points; good: 75–89 points; general: 50–74 points; poor: < 50 points, total 100 points), and the X-ray film of the affected foot was reexamined. A visual analog scale (VAS) was used to evaluate the degree of postoperative pain (painless: 0; mild pain: 1–3 points; moderate pain: 4–6 points; severe pain: 7–10 points). The time to remove the external fixator and the time to remove the internal fixator were determined according to the regular follow-up and evaluation results. If the fracture line was still obvious on X-ray examination, the use time of external fixation was prolonged. If the fracture line was blurred, the brace was changed to external fixation, and ankle joint active and passive nonweight-bearing functional training was started with the company and help of the family. It is worth noting that for children, weight bearing is forbidden for at least 3 months after the operation, and they can only begin to walk and carry out partial weight-bearing activities after the X-ray examination indicates that the fracture line has completely disappeared. The patients then gradually returned to normal activities.

### Research characteristics

The present study has the following characteristics: ① Strict inclusion and exclusion criteria, ensuring the reliability and representativeness of the research. ② Use of the Hawkins classification for talus fractures, a widely recognized and used classification method. ③ All patients underwent anteroposterior and lateral foot X-rays and CT scans, ensuring accurate classification of fracture types. ④ All patients were followed up for at least one year, which helps understand the long-term effects and potential complications after surgery.

### Evaluation methods

Firstly, the clinical effect of the operation was evaluated according to the patient's symptoms and signs, as well as the results of X-ray film and CT examination. Secondly, the use of the American orthopaedic foot and ankle society (AOFAS) after the ankle and foot function scoring system, evaluation of limb function of patients before and after surgery. Finally, any complications that may occur after surgery, such as avascular necrosis of the talus fracture, arthritis and nonunion were recorded and evaluated.

### Data analysis

The patients' sex, age, fracture type, operation time, functional score and postoperative complications were classified. The collected data were processed by SPSS25.0 statistical software, the measurement data were expressed by $\overline{x}\pm s$, and the two independent samples *t* test was used for comparison. The count data were statistically compared by Fisher’s exact test. *P* < 0.05 in the results indicated that the difference was statistically significant.

## Results

### Clinical follow-up results

**Fig. 1:**
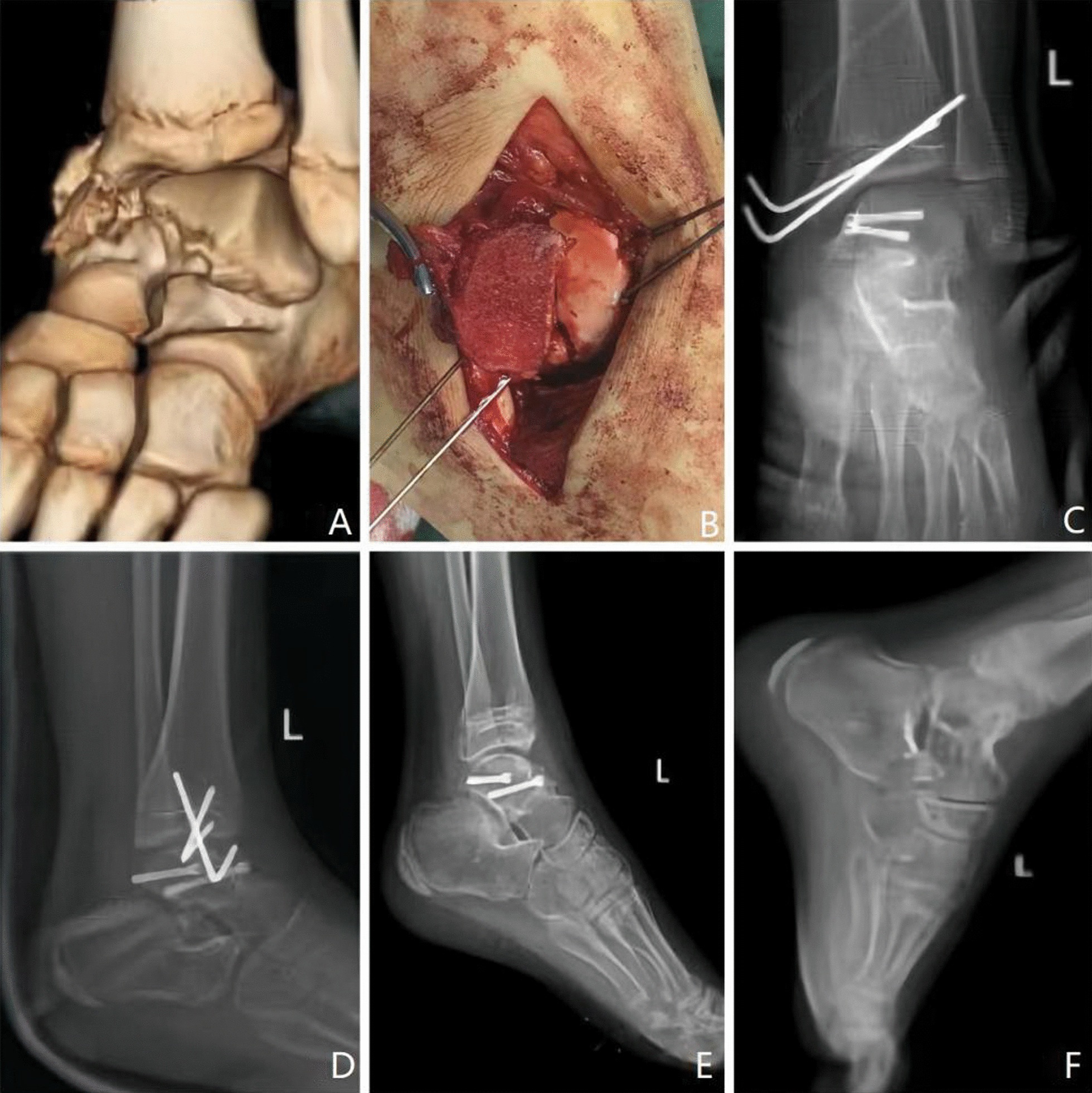
A 7-year-old girl suffered from a left talar fracture caused by a traffic accident. The preoperative CT (**A**) and intraoperative (**B**) images showed a type II fracture of the left talus neck. Postoperative X-ray of the ankle joint (**C**, **D**) showed good reduction in the fracture. At 6 months after the operation, anteroposterior and lateral X-ray films of the ankle joint (**E**) showed that the fracture had healed, and the internal fixation was removed (**F**). At 48 months after the operation, the adduction function of the left ankle joint was worse than that of the contralateral side, and the plantar flexion and dorsiflexion functions were normal. The AOFAS score was 93

All 20 children were followed up for 12 months to 30 months, with an average of 15 months. The incisions of all patients healed with grade I grade A. Two patients had subcutaneous congestion after the operation, and the wounds healed well after active symptomatic treatment and enhanced dressing changes. No postoperative wound infection or skin necrosis occurred during hospitalization. No complications, such as pressure sores, hypostatic pneumonia, or deep venous thrombosis of the lower limbs, occurred. During the later follow-up, 6 cases of talus avascular necrosis were found, including 4 cases of simple avascular necrosis of the talus body and 2 cases of avascular necrosis of the talus with traumatic osteoarthritis and malunion. The postoperative VAS scores of 20 children were as follows: no pain; 14 cases of mild pain, 5 cases of moderate pain, and 1 case of severe pain. The VAS score at the last follow-up showed no pain in 18 cases, mild pain in 2 cases, moderate pain in 0 cases, and severe pain in 0 cases. According to the AOFAS score, 14 cases were excellent, 4 cases were good, and 2 cases were fair, with an overall excellent and good rate of 90%. At the last follow-up, the children with complications had different degrees of abnormal gait or walking pain. After the children were instructed to reduce weight bearing and activities, the abnormal symptoms of the children with avascular necrosis of the talus gradually improved (Fig. 1).

### Influence of different factors on prognosis discussion

**Fig. 2:**
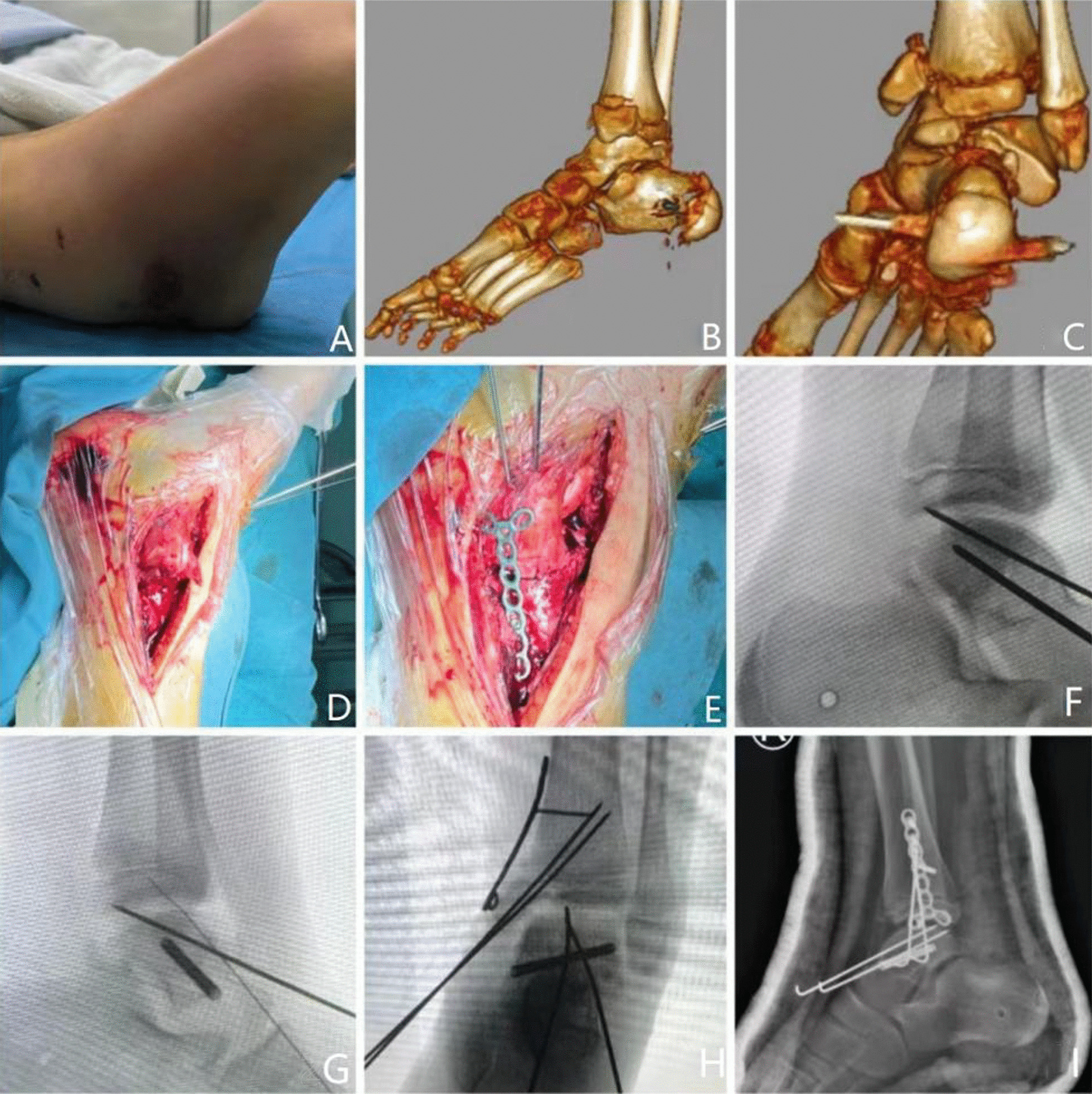
A 9-year-old female patient presented with a right talar fracture caused by a high fall injury. The swelling of the ankle joint gradually disappeared after removing the calcaneal traction device before the operation (**A**). The color film of ankle joint trauma before the operation (**B**, **C**) showed a type II fracture of the right talus neck. Intraoperative reduction under direct vision (**D**, **E**), open reduction and cannulated screw and plate and Kirschner wire fixation were performed. Intraoperative C-arm fluoroscopic ankle joint X-ray (**F**, **G**, **H**) showed good reduction in the fracture. At 2 months after the operation, the patient still received plaster external fixation, and the X-ray (**I**) of the ankle joint showed that the joint space was good, the fracture position was well aligned, and there was no obvious sign of avascular necrosis of the talus neck. The postoperative AOFAS score was 95

During the follow-up, we found that there was no significant difference in the excellent and good rate (76.9%) and necrosis rate (30.8%) between male children and female children (71.4%) and necrosis rate (28.6%) (*P* > 0.05, Table 2). The excellent and good rates (92.9%) of children younger than 9 years old at the time of injury were higher than those of children older than 9 years old (33.3%), and the incidence of avascular necrosis of the talus was lower. The differences between the two groups were statistically significant (*P* < 0.05, Table 3). The average prognosis score of children who underwent surgery within 5 days after injury was 89.2 ± 6.4, which was significantly higher than that of children who underwent surgery after 5 days (72.9 ± 13.1), and the difference was statistically significant (*P* < 0.05). There was no significant difference between patients who underwent surgery within 5 days after injury (15.4%) and those who underwent surgery after 5 days (51.7%) (*P* > 0.05). The data analysis is shown in Table 4. The excellent and good rates of talar neck fracture type II and talar neck fracture type III were 90.1% and 55.6%, respectively. The analysis data are shown in Table 5 (Fig. 2).

**Table 2:** The relationship between sex and prognosis

Gender	Superior	Medium	Bad	Excellent and good rate (%)	Necrosis	Normal	Necrosis ratio (%)
Male	6	4	3	76.9	4	9	30.8
Female	4	1	2	71.4	2	5	28.6
*P*				0.544			0.664

**Table 3:** The relationship between the age of injury and prognosis

Age	Superior	Medium	Bad	Excellent and good rate (%)	Necrosis	Normal	Necrosis
Ratio (%)							
< 9	9	4	1	92.9	2	12	14.3
≥ 9	1	1	4	33.3	4	2	66.7
*P*				0.014			0.037

**Table 4:** The relationship between the timing of surgery and prognosis

Operation opportunity (day)	Sample	AOFAOS measure	Necrosis	Normal	Necrosis ratio (%)
≤ 5	13	89.2 ± 6.4	2	11	15.4
> 5	7	72.9 ± 13.1	4	3	51.7
*P*		0.049			0.122

**Table 5:** The relationship between the type of fracture and prognosis

Type	Superior	Medium	Bad	Excellent and good rate (%)
II	7	3	1	90.1
III	3	2	4	55.6

## Discussion

### Characteristics of talar fractures in children

Talus fracture is a very rare orthopedic disease, with an incidence of less than 1% of all fractures [9]. It has been reported that talar fractures are five times more common in children than in adults [10]. The talus in children is rich in cartilage tissue, which has good elasticity. With increasing age, the proportion of cartilage tissue decreases. The soft tissue and ligaments around the talus have good strength and extension, which makes it have strong impact resistance, so there are more nondisplaced fractures in young children [11]. The talus has an important biomechanical function and plays an extremely key role in the walking and gait of patients. If the fracture cannot be well reduced and fixed, it will seriously affect the normal activity and function of the patient's foot [12]. The injury mechanism of talus fracture is mostly caused by strong violence, such as high fall injury or a traffic accident injury, which causes excessive dorsal extension and compression of the ankle joint, resulting in fracture, separation and displacement [13]. The fragile blood supply and extensive articular surface of cartilage may lead to complications such as avascular necrosis of the talus and osteoarthritis [14]. In this study, there were 12 cases of high falls, 6 cases of traffic accidents and 2 cases of other reasons.

### Classification of talar fractures in children

The talus is divided into three parts: head, neck and body. Nearly half of the entire talus is covered by articular cartilage, without muscle. Because the talus joints have articular surfaces such as the subtalar joint and talonavicular joint, the talus is considered to be an important anatomical feature of a variety of complex joints, including the ankle joint. The talar neck is not only an important structure connecting the head and body but also regarded as a retrograde blood supply channel for the talar body [15, 16]. Most talar neck fractures are caused by axial external forces. This will cause the talar neck to directly impact the anterior edge of the distal tibia and the medial malleolus, resulting in a dorsolateral talar neck fracture. Therefore, the talar neck is prone to varus and plantar flexion deformities, and nearly 28% of patients will also be complicated with medial malleolar fractures [17–19]. Talar head fractures are rare, occurring in 5% to 10% of patients, and are often associated with fractures in other parts of the talus, usually due to the large shear forces in a varus foot or the large compressive forces in plantar flexion acting on the talus head. Talar neck fracture is the most common of the three types of fractures, with an incidence of approximately 50%. At present, talar neck fractures are divided into types I–III according to the Hawkins classification [20], which is the most common method [9, 21]. Type I: no displacement occurred, and the rate of ischemic necrosis in the late stage was less than 10%. Type II: Due to the large displacement of the talus and the subluxation of the talus, the main part of the talus is shifted backward, resulting in varus and valgus of the subtalar joint. This type of fracture causes severe damage to the blood vessels around the neck of the talus and the ligaments; therefore, the incidence of ischemia and necrosis is significantly increased in this type of fracture, ranging from approximately 20% to 50%. Type III is defined as significant displacement, accompanied by dislocation of the tibiotalar joint and subtalar joint and dislocation of the talus body from the ankle hole, resulting in rupture of the posterior bundle of the deltoid ligament and the posterior joint capsule and causing damage to the main blood around the talus. Therefore, the necrosis rate is very high, approximately 80–90%. In 1978, CANALE et al. [22] proposed a new classification of talar neck fracture, type IV, which included talonavicular joint subluxation on the basis of type III, and the incidence of necrosis was close to 100%. Because there is no classification of types with significant displacement but without subtalar dislocation, it has been proposed that further modification of the Hawkins classification is needed. Only type II and type III fractures were included in this study. The incidence of talar body fracture is 13-23%, mainly due to the large axial stress imposed by the distal tibia and calcaneus on the talar body, resulting in talar body fracture. The SNEP-PEN classification is still used for the classification of talar internal fractures [23]: ① Type I, talar internal articular surface fracture; ② Type II, sagittal, coronal and horizontal fractures in the talus; ③ Type III, fracture of the posterior process of the talus; ④ Type IV lateral talus fracture; and ⑤ Type V, comminuted, compressed or ruptured talar body fracture, which has a high rate of necrosis. The prognosis of patients with Type V is very poor.

### Treatment of talar fractures in children

For fractures that are not displaced or are displaced by less than 2 mm, plaster external fixation can be used to fix the ankle in a neutral position for 6 to 8 weeks. On examination of plain radiographs, the fracture line was found to be blurred, after which braces were switched to fixation and functional training without weight bearing being started. After the X-ray film shows disappearance of the fracture line, gradually carrying out weight-bearing exercise can obtain a satisfactory prognosis[24]. Studies have also shown that the rate of late avascular necrosis of the talar body in children with mildly displaced talar fractures is as high as 16%, which is much higher than that in adults. This may be because pediatric patients have a higher rate of misdiagnosis and do not receive effective treatment [25]. For fractures displaced more than 2 mm, open reduction and internal fixation should be performed to promote the blood supply of the talus and reduce the incidence of ischemic necrosis and traumatic arthritis [26]. In addition, during the operation, anatomical reduction should also be performed according to the treatment method of adult intra-articular fractures [7]. There are many surgical approaches for talar fractures. We usually use the anteromedial approach, which can fully expose the talus head, talus neck and most of the talus body. During the operation, it is convenient to reduce the broken end under direct vision, avoiding excessive manipulation to cause damage to local soft tissues. The reduction in most talar fractures can be completed under direct vision. This method is very worthy of promotion. In this study, 20 children with talar fractures were also treated with an anteromedial approach. In addition to open reduction and internal fixation, arthroscopy, as a new minimally invasive surgery, has attracted much attention due to its lower injury and larger exposure area. Because the ankle joint is in the superficial part, it is easy to perforate and locate. It is also easy to design different surgical methods according to the reduction in the fracture to facilitate the operation [27]. In addition, 3D printing technology has been widely used in orthopedic surgery, and it has achieved good results. In terms of operation time, intraoperative blood loss, postoperative functional score, postoperative pain score, and anatomical fracture reduction rate, the use of 3D printing technology significantly improved these factors as compared with the conventional surgery group [28, 29]. Perhaps 3D printing technology will also be a good choice for the treatment of talar fractures in children.

### Timing of surgery for talar fractures in children

Some scholars have noted that the severity of trauma leads to the occurrence of avascular necrosis of the talus, and emergency surgery cannot reduce the occurrence of this condition [30, 31]. Some studies have found that elective surgery does not increase the incidence of complications such as avascular necrosis, but it only affects healing because of infection caused by exposure of the talus due to partial skin lesions [32]. Because talar fractures are often a high-energy multiple injury, it is not convenient to perform a detailed physical examination of children and effectively observe their condition in an emergency. Therefore, some scholars believe that in addition to the severe compression of the adjacent skin and soft tissue and because the open fracture cannot be relieved by manual reduction, the operation should be postponed until the soft tissue swelling subsides [33]. In our study, 13 patients were operated on within 5 days, and 7 patients were operated on after 5 days. However, there was no statistically significant difference in the incidence of femoral neck necrosis between the two groups.

### Complications of talar fractures in children

There are many complications of talar fractures in children, among which the incidence and severity of talar avascular necrosis are the largest, especially talar neck fractures. There are also some other complications, such as malunion of talar neck fracture and traumatic osteoarthritis. Some scholars have studied this, and the results show that there is no statistically significant difference in the necrosis rate of the talar body and talar neck after fracture (*P* > 0.05). When the talar body is comminuted and severely displaced, the necrosis rate increases significantly [34]. Therefore, most scholars agree that the incidence of avascular necrosis of the talus after fracture is related to the degree of displacement of the broken end [31, 32, 35]. Malunion has been classified into the following types: type I, malunion or displacement of the talus; type II, talus nonunion with displacement; type III, partial avascular necrosis of the talus on the basis of type I or II; type IV, avascular necrosis of the entire talus on the basis of type I or II; and type V, infectious talar necrosis occurs on the basis of type I or II. In terms of treatment, open reduction and internal fixation can be used for types I and II, while arthrodesis can be used for types III and IV [36]. In our study, ischemic necrosis of the femoral neck occurred in 6 patients. One study found that the incidence of ischemic necrosis of the femoral neck was 0 in children under 12 years old and 31.3% in those older than 12 years old. The researchers also found that although the mechanism of injury was similar, fractures were more severe in adolescents. This suggests that at 12 years of age, if the fracture has been completely displaced, surgical treatment may be considered [37]. Studies have shown that the incidence of talar neck fracture malunion is approximately 20% to 37%, and talar neck varus malunion is the most common type. This is mainly because dorsomedial comminution often occurs in talar neck fractures, which, if not treated correctly and effectively during surgery, will lead to varus deformity, which in turn will lead to biomechanical changes in the subtalar and talonavicular joints and eventually lead to posttraumatic osteoarthritis [19]. Traumatic osteoarthritis is the most common complication of femoral neck fracture, with an overall incidence of 38–49%, and the subtalar joint is the most commonly affected site, with an incidence of 50–100% [38, 39]. Therefore, making a detailed preoperative plan is the key to avoiding postoperative complications of talar neck fracture.

### Study limitations

Talus fracture in children is a rare bone disease, and there is no effective treatment in the clinic. There are many complications of late healing, which have a great impact on the quality of life and motor ability of children in the future. Because of the small number of cases, there is no generally accepted treatment for this disease, and it is not clear how different clinical factors affect the outcome of this disease. Therefore, for future research, we should begin from the following aspects. First, we should concentrate more cases and conduct large-scale multicenter studies to determine the most effective treatment. Secondly, the pathophysiological mechanisms of talar fractures should be thoroughly studied to discover possible new therapeutic targets. Third, long-term follow-up studies should be considered to evaluate the long-term effects and possible complications after treatment. Finally, randomized, controlled trials of different treatment approaches should be conducted to determine which is most effective and safe. It is hoped that these recommendations will provide some direction for future research and ultimately lead to better treatment outcomes for patients.

## Conclusion

Using the anteromedial approach combined with nail fixation offers distinct advantages in treating Hawkins II/III talus fractures in children. Firstly, this method provides surgeons with a clear surgical view. Every step in the surgical process, from fracture reduction to fixation, can be executed under direct vision. This significantly improves the accuracy and success rate of the surgery. Secondly, this technique does not affect the stability of the ankle joint. The stability of the ankle joint is particularly important for children, as they require a stable joint to support bodily activities and movements during their growth. With this method, doctors can ensure that while treating the talus fracture, the stability of the ankle joint remains uncompromised. Additionally, this approach aids in the recovery of ankle joint function. After surgery, as the fracture heals, children can gradually resume normal ankle movements under the guidance of a doctor. This technique ensures that the ankle joint maintains good functionality during the post-surgical recovery period. In conclusion, using the anteromedial approach combined with nail fixation to treat Hawkins II/III talus fractures in children is a highly effective method. It not only offers a clear surgical view and guarantees surgical precision but also aids in the recovery of ankle joint function, making it a reliable surgical option for treating talus fractures in children.

## Data Availability

All data generated or analyzed during this study are included in this published article.
